# DNA Repair Gene Expression Adjusted by the *PCNA* Metagene Predicts Survival in Multiple Cancers

**DOI:** 10.3390/cancers11040501

**Published:** 2019-04-08

**Authors:** Leif E. Peterson, Tatiana Kovyrshina

**Affiliations:** 1 Department of Healthcare Policy and Research, Weill Cornell Medical College, Cornell University, New York City, NY 10065, USA; 2 Center for Biostatistics, Institute for Academic Medicine, Houston Methodist Research Institute, 6565 Fannin Street, Houston, TX 77030, USA; tkovyrshina@houstonmethodist.org

**Keywords:** RNA-seq, oncology, DNA repair, survival, *PCNA* metagene

## Abstract

Removal of the proliferation component of gene expression by proliferating cell nuclear antigen *(PCNA*) adjustment via statistical methods has been addressed in numerous survival prediction studies for breast cancer and all cancers in the Cancer Genome Atlas (TCGA). These studies indicate that the removal of proliferation in gene expression by *PCNA* adjustment removes the statistical significance for predicting overall survival (OS) when gene selection is performed on a genome-wide basis. Since cancers become addicted to DNA repair as a result of forced cellular replication, increased oxidation, and repair deficiencies from oncogenic loss or genetic polymorphisms, we hypothesized that *PCNA* adjustment of DNA repair gene expression does not remove statistical significance for OS prediction. The rationale and importance of this translational hypothesis is that new lists of repair genes which are predictive of OS can be identified to establish new targets for inhibition therapy. A candidate gene approach was employed using TCGA RNA-Seq data for 121 DNA repair genes in 8 molecular pathways to predict OS for 18 cancers. Statistical randomization test results indicate that after *PCNA* adjustment, OS could be predicted significantly by sets of DNA repair genes for 61% (11/18) of the cancers. These findings suggest that removal of the proliferation signal in expression by *PCNA* adjustment does not remove statistical significance for predicting OS. In conclusion, it is likely that previous studies on *PCNA* adjustment and survival were biased because genes identified through a genome-wide approach are strongly co-regulated by proliferation.

## 1. Introduction

Genomic instability is an important hallmark of cancer which leads to mutations that dysregulate cellular growth [1,2]. Mutations play an important role in oncogenic transformation and can be catastrophic during mitosis [3,4]. The additional replication stress and increased oxidative damage that arises from continuous forced cell division in tumor cells requires several DNA repair components [5,6]. However, inherited genetic polymorphisms and oncogenic loss result in DNA repair deficiencies, and therefore alternative DNA repair pathways must be found if replication is to continue [7]. The addiction to alternative DNA repair pathways by cancer can therefore be targeted to prevent the repair and restart of stressed replication forks [8,9,10].

All living cells depend on DNA repair to correct damage to DNA, which can arise from mismatched bases, oxidized bases, DNA methylation, DNA adducts, intra- and interstrand DNA crosslinks, single-strand and double strand breaks, and stalled forks. DNA repair pathways include direct reversal repair, base excision repair, non-homologous end-joining, mismatch repair, translesion synthesis, damage signaling, homologous recombination, and nucleotide excision repair. Early attempts to treat cancer focused on inducing DNA damage by introducing an excess of lethal mutations through radiotherapy and chemotherapy, which initially resulted in cellular death. Unfortunately, it was observed that tumor cells repaired therapy-induced DNA damage [11,12]. What is surprising is that, while tumor cells can be initially repair deficient as a result of inherited genetic polymorphisms or acquire repair deficiency from oncogenic loss, they can nevertheless find alternative repair pathways to survive and can correct therapy-induced DNA damage. As a result, current repair-centric therapies target the inhibition of alternative repair mechanisms, known as “synthetic lethality” to increase sensitivity to traditional therapeutics [13,14].

Recently, it was reported that the proliferating cell nuclear antigen (*PCNA*) DNA repair protein is widely co-regulated in the genome [15,16]. *PCNA* is a ring-like protein which serves as a co-factor for polymerase δ, and surrounds DNA during strand synthesis to recruit proteins needed for DNA replication and repair [17]. *PCNA* by itself is not a tumor suppressor gene or oncogene, but rather is a proliferation promoting protein whose expression is upregulated during cell replication. Ge et al. [18] identified 131 mRNAs in 36 types of normal tissues whose expression correlated *r* > 0.65 with expression of *PCNA*. Expression patterns of these 131 genes were collapsed into a median value (called *PCNA* “metagene”) by Venet et al., who removed the proliferation effect by performing multivariate analysis of expression profiles published by 47 breast cancer (survival) studies [16]. Their results indicated that 91% of the genes predictive of survival were significantly correlated with the *PCNA* metagene, which removed the statistical significance that was reported by the original studies. Random bias, which occurs when randomly selected genes predict overall survival (OS) with greater significance, was also observed by Venet et al. [16] in most of the studies considered. Shimoni also investigated *PCNA* adjustment of RNA-Seq expression during survival analysis for 34 cancers from TCGA and reported that *PCNA* adjustment removed statistical significance for OS prediction and resulted in random bias [19].

While genome-wide approaches have revealed that *PCNA* adjustment to expression removes statistical significance for survival prediction, a hypothesis-driven approach specifically focusing on removal of the proliferation component of expression in DNA repair genes used for survival analysis has eluded systematic investigation. We therefore pursued a candidate gene approach to determine survival prediction with DNA repair gene expression adjusted for *PCNA* using TCGA RNA-Seq data. The hypothesis was that *PCNA* adjustment would not remove a significant association between expression of DNA repair genes and OS. The rationale and importance of this translational hypothesis is that new lists of repair genes which are predictive of OS can be identified to establish new targets for inhibition therapy. Not all of the cancers available in TCGA were investigated because the body of information on DNA repair inhibition therapy is more strongly hinged to common cancers [14,20,21]. Another hypothesis was that patterns of somatic mutations, deletions, and amplifications in cancer-specific driver genes and the DNA repair genes considered would provide new insight into the patterns of genomic alteration observed in tumor cells [22]. Results of the computational analyses were used for generating lists of DNA repair genes whose upregulation was associated with shortened survival, which would be potentially amenable for inhibition therapy to prolong survival.

## 2. Data

### 2.1. Cancer Data

The data used in this investigation were derived from genomic sequencing of tumors in TCGA [23]. We investigated DNA repair gene expression in 18 cancers for which genomic sequencing and RNA-seq expression data were available from cBio-Portal (http://www.cbioportal.org) [24,25]. Specifically, the cancers for which only age at diagnosis was available, and not pathological stage, in the TCGA data were acute myelogenous leukemia (AML), bladder, low grade gliomas, glioblastoma multiforme (GBM), head and neck, and sarcoma. Cancers for which both age at diagnosis and pathological stage were available included breast, cervical, colorectal, liver, lung, lung squamous cell, melanoma, ovarian, renal clear cell, renal papillary, stomach, and uterine. Altogether, this resulted in a total of 18 cancers that were considered.

### 2.2. Expression, Mutations, Deletions, and Amplifications

RNA-Seq based normalized expression values for somatic mutations in DNA repair genes were also obtained from cBio-Portal [24,25]. We also acquired high-confidence deletions and amplifications from cBio-Portal, where a deletion was defined as full homozygous loss with a GISTIC score [26] of −2, and an amplification was defined as high-level gain with a GISTIC score of 2. Low-level deletions (heterozygous loss) and low-level gain (low-level amplifications) with GISTIC scores of −1 and 1, respectively, were not used.

### 2.3. DNA Repair Gene Lists

We used the comprehensive list of DNA repair genes provided by Wood et al. [27], which have been previously described [28,29,30,31,32,33]. Table 1 lists the DNA repair pathways and 123 genes used which were available in TCGA expression data. 

A brief description of each pathway follows. Direct reversal DNA repair (DRR) is a single step reaction of removal of the methyl- or photoadducts. Base excision DNA repair (BER) corrects base lesions generated by oxidative, alkylation, deamination, and depurinatiation/depyrimidination damage. Non-homologous end-joining (NHEJ) repairs double-strand breaks (DSBs) at all stages of the cell-cycle, bringing about the ligation of two DSBs without the need for sequence homology, and therefore NHEJ is error-prone. DNA mismatch repair (MMR) is responsible for correction of replication errors (mismatches, small insertions, deletions, and microsatellites) that escape the proofreading activity of a DNA polymerase [34]. Translesion synthesis (TLS) is a process involving specialized DNA polymerases which replicate across from DNA lesions. DNA damage signaling (DDS) induces several cellular responses including DNA repair, cell-cycle checkpoint activity, and triggering of apoptotic pathways. Homologous recombination repair (HRR) is a type of genetic recombination in which nucleotide sequences are exchanged between two similar or identical molecules of DNA. Nucleotide excision repair (NER) removes UV-induced damage (thymine dimers and 6-4-photoproducts) as well as other kinds of DNA damage, which produce bulky distortions in the shape of a DNA double helix.

## 3. Results

### 3.1. Cancer Survival Prediction—Univariate

**Figure 1:**
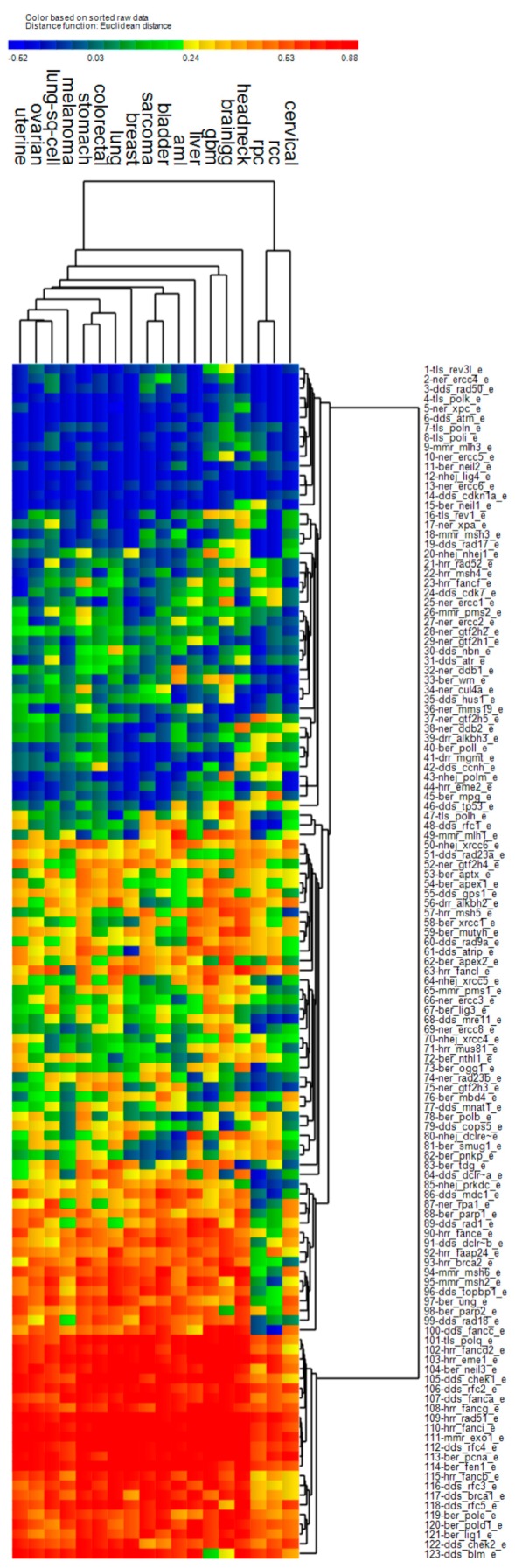
Pearson correlation between expression of 123 DNA repair genes (adjusted for age at diagnosis) and *PCNA* metagene expression (median expression of 131 genes correlated with *PCNA*) within the 18 cancers considered. Heat map colors represent negative correlation (blue) and positive correlation (red). RCC: renal clear cell cancer; RPC: renal papillary cancer.

To understand the effect of the *PCNA* metagene on OS prediction by repair genes, we first estimated Pearson correlation between each DNA repair gene’s expression (adjusted for age at diagnosis) with expression of the *PCNA* metagene. Figure 1 shows a cluster heatmap for the 123 repair genes and each of the 18 cancers considered, with blue-coloring denoting low correlation with the *PCNA* metagene and red indicating high correlation with the *PCNA* metagene. Renal clear cell (rcc) and renal papillary cancer (rpc) clustered together along with cervical cancer in their own “tree branch” of the dendogram, because the two cancers had much lower correlation (blue) with the *PCNA* metagene when compared with all remaining cancers (red). Head and neck cancer clustered by itself as an outlier, because repair gene correlation with the *PCNA* metagene was greater than other cancers for genes 94–123 (red color). Glioblastoma multiforme (gbm) and brain low-grade gliomas (brainlgg) clustered together mostly because of shared correlation of repair genes and the *PCNA* metagene for these cancers, and the unique pattern involving lower correlation between *BLM* (gene 123) expression and the *PCNA* metagene. The remaining cancers showed similar correlations between repair genes and *PCNA*, and therefore clustered together with slightly varying patterns. The biological importance of the correlation shown reveals how each repair gene is potentially coregulated by proliferation, which may or may not be important for readers developing models of repair gene co-regulation by *PCNA*. Certainly, the associations reported would need to be verified experimentally.

**Figure 2:**
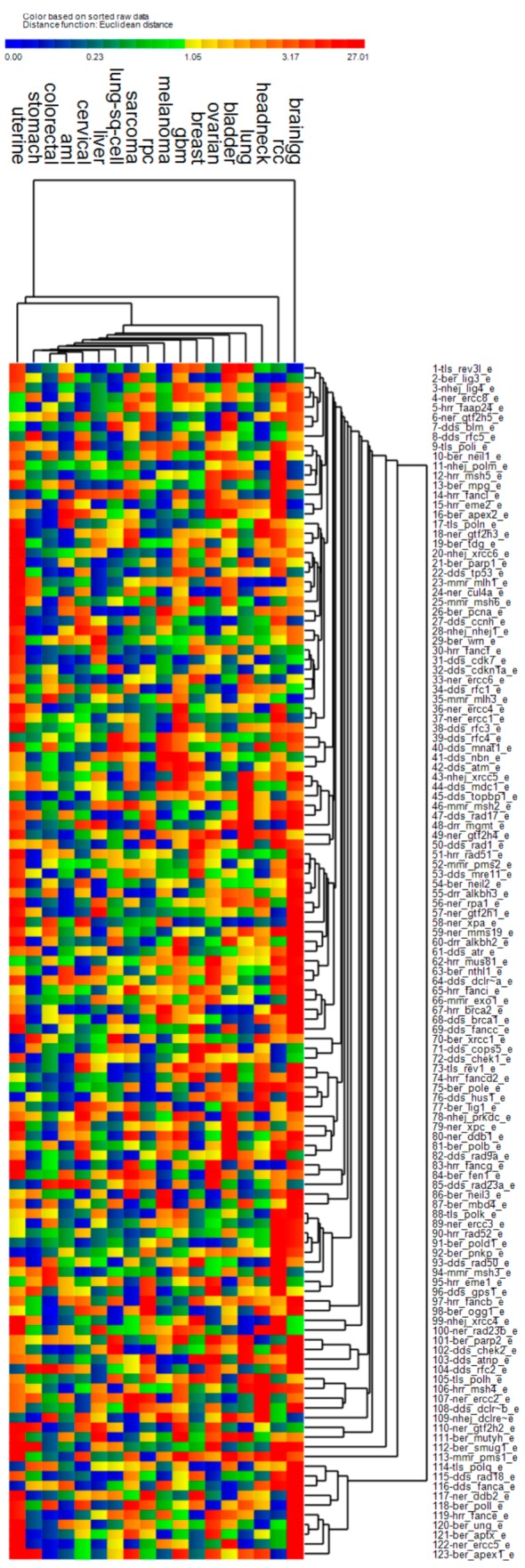
Chi-squared test statistics from Kaplan-Meier (KM) tests based on DNA repair gene expression adjusted for age and stage at diagnosis, and the *PCNA* metagene. Gene-specific adjusted expression values were split into two groups based on a median cut-point. Heat map colors represent chi-squared values for each KM test, where blue-green-yellow-orange denotes non-significant tests and red denotes significant tests, i.e., χ^2^ > 3.84.

Next, we report the univariate Kaplan-Meier (KM) test results for each gene based on expression adjusted for age at diagnosis, stage, and the *PCNA* metagene. Figure 2 illustrates the KM chi-squared values (χ^2^) for each gene when used to predict OS for the various cancers. Overall, most of the genes were not significant predictors of OS, since significant KM tests are required to have χ^2^ > 3.84 (red color). Low-grade gliomas (brainlgg), renal cell cancer and uterine cancer clustered by themselves as individual outliers since they had many more genes which predicted OS significantly (red). The remaining cancers had different patterns of χ^2^, suggesting uniqueness of the cancer in terms of OS prediction by repair genes. Previous reports [1,2] have suggested that adjustment of genome-wide transcript expression with the *PCNA* metagene before survival analysis reduces the chance of finding significant predictors of OS. However, when considering DNA repair genes, there appears to be many cancer-repair gene combinations for which OS can be predicted significantly when removing proliferation from expression.

### 3.2. Cancer Survival Prediction—Multivariate Gene Sets for Multiple Pathways

In this section we report on multivariate sets of genes which were significant predictors of OS. Two types of hypothesis test results are provided: one in which only KM tests were used and the other based on randomization tests for OS in which same-size gene sets were randomly selected from the pool of DNA repair genes. 

Table 2 lists sample sizes and cancer sites for which KM logrank tests of OS based on the best binarized principal components (PC) from correlation of multiple DNA repair gene expression adjusted for age and the *PCNA* metagene. For the simultaneous adjustment of expression by age and *PCNA* metagene (first A,P column), all of the cancers resulted in a significant KM logrank test for the best binarized PC; however, the empirical *p*-values for random selection of genes resulted in a significant KM test (second A,P column) for three cancers, namely, AML, bladder, and sarcoma.

Table 3 lists sample sizes and cancer sites for KM tests performed on the best binarized PC for multiple DNA repair gene expression adjusted for age, stage, and the *PCNA* metagene. For the simultaneous adjustment of expression for age, stage, and *PCNA* metagene (first A,S,P column), all of the 12 cancers resulted in PCs whose KM test result were significant. However, when random gene sets of the same size were selected, (second A,S,P column), only eight of the 12 cancers were significant: breast, colorectal, liver, lung, lung squamous cell, melanoma, renal papillary cancer, and stomach. The combined results in Table 2; Table 3 suggest that while age, stage, and *PCNA* metagene adjusted expression of DNA repair gene expression resulted in significant KM tests for 100% of the cancers (18/18), randomization tests were significant for 61% (11/18) of the cancers. 

This is important because it supports our hypothesis that *PCNA* adjustment does not remove statistical significance for predicting OS when using DNA repair genes. 

### 3.3. Multivariate Sets of DNA Repair Genes which Predict Overall Survival for each Cancer

This section describes for each cancer the multivariate list of genes constructed from individual KM tests using PCA. The loadings on the best binarized PCs with the greatest significance for predicting OS based on a KM test were used to reveal whether downregulation or upregulation resulted in shortened or prolonged OS. Table 4 lists the DNA repair genes of the best binarized PC for 10/11 cancers showing empirical *p*-values less than 0.05 when adjusting for age, stage, and *PCNA* metagene. The gene set for liver cancer is not shown because there were 43 genes which loaded on the best binarized PC, suggesting the model is not parsimonious. 

When considering the composite of all the genes which were significant survival predictors, pathway activation results indicate upregulation of the *BRCA1* pathway, NHEJ pathway, BER pathway, MMR pathway, and downregulation of the NER pathway. *ATM* signaling (*ATM*, *RAD17*, *RAD50*) was downregulated due to strong downregulation of *ATM*, which one possible mechanism has been suggested to be promoter hypermethylation [35]. With downregulation of the tumor suppressor kinase *ATM* and the checkpoint kinase *CHEK2*, it follows that the *ATM* intraphase checkpoint would also likely be inactivated. Cells deficient in *ATM* have also been observed to not reduce transcription following DSBs, a phenotype which has been called “radiosensitized DNA synthesis”. In addition, ataxia-telangiaectasia (A-T) cells deficient in *ATM* are known to repair DSBs following exposure to ionizing radiation [36]. With regard to the *BRCA1* pathway, Complex B was highly upregulated with downstream upregulation of G1/S-Phase as well as the HRR pathway (*BLM*, *BRCA1*, *MSH2*, *MS62*, and *RFC*). In addition, *FANCD2* was upregulated, which activates S-Phase checkpoint control. However, Complex C was downregulated (mostly due to *RAD50*), possibly suggesting downregulation of downstream G2/M phase. In the NHEJ pathway, *ATM*, the cross-linking enzyme Artemis (*DLCRE1C*), and *RAD50* were downregulated, however, *LIG3*, *LIG4*, *NBN*, *PRKDC*, *XRCC1*, *XRCC2*, and *XRCC5* were upregulated. Activated genes in the BER pathway included *POLB*, *POLE*, *XRCC1*, *LIG1*, *LIG3*, and *FEN1*. The MMR pathway activated genes were *EXO1*, *FEN1*, *MSH2*, *MSH6*, *RFC2*, and *RFC3*. Downregulated genes in the NER pathway were the sensitizer *APEX1* and the DNA glycosylate *OGG1*. The NER pathway was mostly downregulated due to downregulation of *HR23B*, *TFIH*, *XPC*, and *XPG*. We also noted that *MGMT* was downregulated, which would increase SSBs and their conversion to DSBs at replication forks [37]. Another observation was that *PARP1* was not in any of the lists of significant genes, because from a univariate perspective, it correlated positively with the *PCNA* metagene in all cancers except renal clear cell and renal papillary (see gene #88 in Figure 1). *PARP1* was also a weak predictor of OS for most cancers after its expression was adjusted for age at diagnosis and the *PCNA* metagene (see gene #21 in Figure 2).

### 3.4. Cluster Analysis of Genomic Event Rates

**Figure 3:**
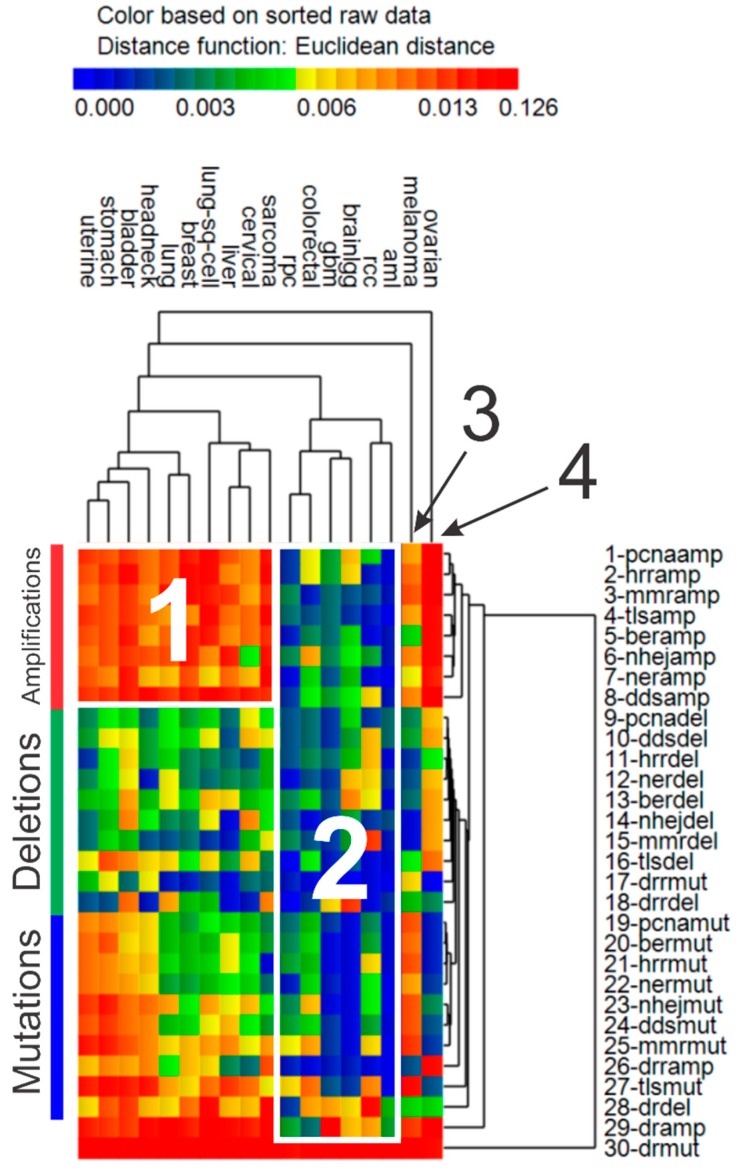
Genomic event rates (GER). Hierarchical cluster analysis results of GER (per tumor-gene) for somatic mutations (“mut”), deletions (“del”), and amplifications (“amp”) in driver genes (“dr”) and the 8 groups of DNA repair genes (DRR, BER, NHEJ, MMR, TLS, DDS, HRR, NER). Euclidean distance used as the distance function, with unweighted pair group method with arithmetic mean (UPGMA) as the agglomeration method. Heat map colors represent GER.

Once genomic event rates (GERs) were determined for each cancer and the sets of driver genes and DNA repair genes in each repair pathway, cluster analysis was pursued to portray the pattern of similarity between the various cancers based on GERs. Results of cluster analysis of GERs for various cancers is shown in Figure 3. A total of four clusters of cancers were discernible in the data. In spite of all the cancers exhibiting high GERs for driver mutations, cancers in cluster 1 portray strong upregulation of genomic amplification in DNA repair genes, while cancers in cluster 2 reveal downregulation of amplification, deletion, and mutation in DNA genes. Melanoma and ovarian cancer clustered furthest away from the previously described clusters, mostly because of the unique patterns among GERs which emerged. Regarding driver genes, both melanoma and ovarian cancer exhibited greater rates of amplifications but had lower rates of deletions. Additionally, while melanoma revealed increased rates of mutations and amplifications and decreased rates of deletions in DNA repairs genes, ovarian cancer showed the opposite pattern, with lower rates of mutations in DNA repair genes and greater rates of amplifications and deletions. Table 5 lists qualitative patterns which emerged from the cluster analysis of GERs shown in Figure 3.

Figures S1–S36 illustrate for each cancer investigated a KM plot and Kernel density (pdf) plot of *p*-values during random selection of gene sets for the best binarized PCs derived from sets of genes, for which each gene had its own significant KM test after the various adjustments for age, stage, and *PCNA* metagene. *p*-Values listed in Table 2 and Table 3 were extracted from Figures S1–S36.

## 4. Discussion

Cancer is a multifactorial disease which depends on a constellation of factors involving genomic instability, selective genetic pressure from somatic mutations and polymorphisms, and gene-environment interactions. Two important hallmarks of cancer are the persistent high level of somatic mutations in driver genes and DNA repair addiction. Together, these mechanisms directly and indirectly support a growth advantage and prolonged cell survival. As the costs of genomic DNA sequencing and RNA-Seq analysis decrease, there will continue to be new information available regarding cancer’s addiction for DNA repair.

Our approach employed two levels of statistical testing, one that merely involved straightforward maximum likelihood (ML) estimation and another based on randomization tests, which resulted in empirical *p*-values. The ML-based survival prediction with adjusted DNA repair gene expression was significant for most of the cancers; however, survival prediction based on empirical *p*-values was significant for fewer cancers. It is now known that identification of sets of genes from genome-wide annotation lists will result in false positives that are associated with the *PCNA* metagene. Our focus was to specifically target DNA repair gene expression, remove the effect of the *PCNA* metagene, age at diagnosis, and stage, to determine if significant lists can be obtained. Not surprisingly, after the adjustments, many of the cancers revealed DNA repair genes which significantly predicted OS.

It is important to realize that our use of randomly selected same-size gene sets was performed in order to develop randomization tests for empirical *p*-value testing. Since the study was hypothesis-driven and used a candidate gene approach with a constrained list of DNA repair genes, it would be impossible to determine the effects of random bias based on randomly selecting genes from a genome-wide perspective as was done by Venet et al. [16] and Shimon [19]. There may likely be genes in the genome which predict survival better than the DNA repair genes considered; however, they would not be DNA repair genes. There was also no freedom to use genome-wide selection of genes for survival prediction, due to our candidate gene approach.

Pathway analysis results indicate a pattern suggestive of downregulation of primary damage signaling kinase (*ATM*) and initial BER pathway components (*APEX1* and *OGG1*), and when combined with increased pathogenic somatic mutations in driver genes (e.g., TP53), our results may indicate that damage signaling in the initial portion of repair pathways is abrogated, while the remainder of the pathway is intact.

We also assessed GERs of the various cancers and confirmed that all of the cancers had high somatic mutation rates in driver genes. There were also two main clusters of cancers identified, which portrayed either high levels of amplification in DNA repair genes or low GERs for mutations, deletions, and amplification in DNA repair genes. The latter group of cancers including AML, colorectal, GBM, low grade gliomas, and renal papillary (cluster 2 in Table 5), may be more opportunistic for repair inhibition therapy because of less confounding associated with low levels of mutations, deletions, and amplification exhibited in DNA repair genes for these cancers. It warrants noting that the heat map in Figure 3 represents cancer-specific GERs in DNA repair pathways and not genome-wide mutations levels in cancers as reported by Lawrence et al. [38]. While melanoma was reported by Lawrence et al. to have the greatest levels of genome-wide mutation levels, our results indicate that melanoma had high levels of gene amplification and somatic mutations in DNA repair genes, causing its separate clustering. In addition, ovarian cancer clustered by itself because of high levels of amplification in DNA repair genes.

Random somatic mutations considered in TCGA data are not genetic polymorphisms (e.g., SNPs) occurring in the same regions of DNA, for which allelic phenotypes, associated risks, prognosis, and recommended treatment options are known. Instead, they are rarely found in the same location in DNA and are rarely of the same type. Somatic mutations merely accumulate from genetic selective pressure in driver genes, which is one of the most important hallmarks of sporadic cancers. Along these lines, there is uncertainty related to experimentally verifying the effect and pathogenicity of a single somatic mutation. Numerous algorithms can be employed for computationally predicting pathogenicity of somatic mutations (our results are based on the FATHMM algorithm used by TCGA and COSMIC [39]), but the experimental laboratory costs required to fully understand how a single somatic mutation alters a protein and how any change in function impacts the disease phenotype are exorbitant.

The translational value of our results is established by the potential of novel patterns of DNA repair gene expression in cancer, which could prove useful in animal studies, transgenics, and xenograft models, etc., in order to understand if inhibition of the genes identified inhibit tumor growth and improve survival [40,41,42]. Adjustment of DNA repair gene expression by the *PCNA* metagene has enabled us to view cancer from a distant perspective based on high-granularity involvement of DNA repair pathways in cancer. This view will hopefully enforce an appreciation among biologists and oncologists for the translational value of pursuing experimental inhibition studies, as well as randomized control trials for establishing safety and evaluating efficacy.

We did not comparatively assess numerous techniques for their computational efficiency, scalability, or differences in OS survival prediction. We also did not evaluate differences between using progression free survival (PFS) vs. OS, or bootstrapping effects on results. TCGA was primarily undertaken for molecular studies, and therefore clinical data standardization and collection was a secondary effort [43]. TCGA was also not a clinical trial, and therefore outcomes were considered from both retrospective and prospective cases, without a standardized patient follow-up plan. Molecular data were also obtained from single sections of primary tumors, and therefore spatial and temporal variation in tumor heterogeneity cannot be addressed. Use of OS as a survival endpoint is supported for most of the cancers available in TCGA [43]. For breast cancer subtypes with varying aggressiveness, OS is likely appropriate for the basal-like subtype but not for luminal A. Glioblastoma multiforme is also considered an aggressive cancer, although OS is considered suitable for use with TCGA data. Prostate cancer OS in TCGA is not a suitable endpoint, and therefore prostate cancer was not evaluated. Regarding confounding, we removed the effects of age and stage at diagnosis from expression data using skew-zero inputs to multivariate regression. Confounding caused by competing risks and outcomes in TCGA is more relevant for disease-specific survival (DSS), disease-free interval (DFI), and PFS and less of an issue for OS, so we do not envision a sufficient level of competing risks bias which would trigger a concern. The Cox PH assumption is also supported for most of the TCGA cancers, with only a few exceptions. While KM analysis was employed as the primary survival hypothesis test, Cox PH regression was only used for determination of risk directionality as a function of increasing expression level.

There is also the problem of unknown upstream effects of germline polymorphisms and DNA repair deficiencies which may result in a variety of unknown influences. The difficulty presented by cellular niching and high levels of clonal heterogeneity in tumors also presents a challenge for fully unraveling the associations observed in this study. The TCGA data used are not based on single-cell RNA-Seq analysis, which would be helpful for elucidating heterogeneity effects; however, the large variation in genotypes would exacerbate the present uncertainties surrounding our attempt to portray the role of DNA repair genes in cancer survival. We also did not employ empirical data from TCGA for DNA microsatellite instability, methylation status, or chromosome aberrations, which would overlay more complexity on the models developed. Although we did include DNA repair genes in the MMR pathway, which verify repeat count of microsatellites during cell division [44].

## 5. Materials and Methods

### 5.1. PCNA Metagene

Table S1 of the Supplemental Information lists the 131 *PCNA*-related genes employed for *PCNA* adjustment. For each tumor, we obtained RNA-Seq derived normalized expression values of the 131 *PCNA*-related genes [16], and collapsed their expression values down to median expression, which is termed the “PCNA metagene”. Next, the *PCNA* metagene (median) and expression values for all of the DNA repair genes listed above were transformed into van der Waerden (VDW) scores. This transformation simply involved first transforming expression values for each gene into percentiles, and then substituting the percentiles as probabilities in the inverse standard normal function, i.e., Z = Φ^−1^(*ptile*), to obtain standard normal variates of expression, which are distributed with mean zero and variance unity.

### 5.2. Maximum Likelihood Survival Prediction

**Figure 4:**
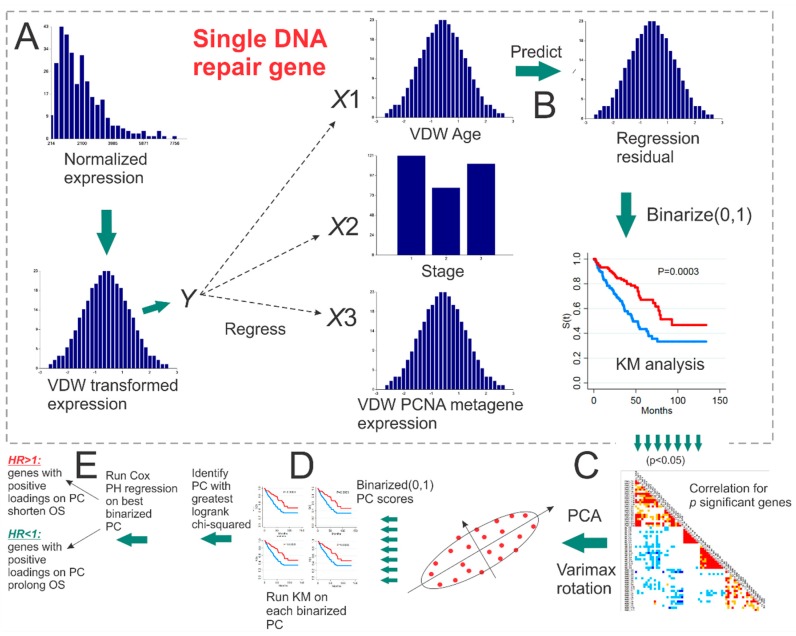
Workflow for identifying the “best binarized PC” for a set of significant genes from univariate Kaplan-Meier analyses. (**A**) van der Waerden (VDW) scores of log-transformed expression values for each DNA repair gene are regressed on the VDW scores for age at diagnosis, stage, and the *PCNA* metagene. (**B**) The residual values from each linear fit (i.e., expression with the effect of age at diagnosis, stage, and *PCNA* metagene removed) are then binarized (0,1) and input as the univariate grouping value during Kaplan-Meier (KM) analysis of overall survival (OS). (**C**) PCA with Varimax orthogonal rotation is performed on the correlation matrix of *p* significant binarized residual vectors. (**D**) Each of the *p* principal component (PC) score vectors is binarized (0,1) and input into univariate KM analysis. (**E**) The PC resulting in the greatest KM chi-squared value is selected as the “best binarized PC,” and univariate Cox PH regression is then run on the best binarized PC to determine if positive (negative) PC score values are associated with prolonged (shortened) OS.

The VDW scores for expression of each DNA repair gene were regressed separately on the VDW scores for age, VDW scores for tumor stage, and VDW scores for *PCNA* metagene, and the residuals were taken as the new DNA repair gene expression values. Residuals for each DNA repair gene were then binarized into (0,1) by splitting on the median, to form a grouping variable which was employed in Kaplan-Meier (KM) survival analysis with OS status. Overall survival was reported to be the most accurately derived outcome for TCGA data [43]. Each DNA repair gene which resulted in significant maximum likelihood (ML) estimates of the KM logrank tests, i.e., χ^2^ *p*-value < 0.05 (1 d.f. chi-squared), was appended to a list of *p* genes. Eigendecomposition of the correlation matrix of the *p* significant genes was then performed with Varimax orthogonal rotation, and the PCs for all *p* dimensions were extracted. Each PC was then transformed into a binary grouping variable for KM input, by assigning negative PC values to 0 and positive to 1. The single group-transformed PC which resulted in the greatest χ^2^ value during KM analysis was identified and called the “best binarized PC”. For cancers without stage available in TCGA, the best binarized PC when age and *PCNA* were used for residual generation was input into Cox proportional hazards (PH) regression as a continuous variable (i.e., PC score) to determine whether positive values prolonged or shortened OS. Whereas, for cancers with stage available in TCGA, the best binarized PC when age, stage, and *PCNA* were used for residual generation was input into Cox PH regression. For the best binarized PC under evaluation, if the Cox PH hazard ratio (*HR*) < 1, then genes having positive loading on the best binarized PC were beneficial to OS if upregulated, whereas genes whose loadings were negative were considered hazardous, and would need to be downregulated in order to be beneficial. Analogously, if the Cox PH *HR* > 1, it meant that positive PC values were deleterious, and therefore genes that loaded negatively on this PC would need to be upregulated to be beneficial, and genes that loaded positively on this PC would need to be downregulated in order to be beneficial to OS. Figure 4 illustrates the workflow employed, outlining the various steps used for establishing the best binarized PC for each cancer, and whether positive loadings on the best binarized PC prolonged or shortened OS. Justification for using KM analyses was hinged to our observation that for the same genes, results from Cox PH regression of continuously-scaled expression were consistently more significant when compared with grouped analysis based on KM. Thus, we chose KM analysis for prediction significance due to conservativeness, and Cox PH to determine directionality of survival risk as a function of increasing expression values. Table S2 lists the multiple linear regression results for each DNA repair gene (dependent variable) and Z-scores for the independent variables: age, stage, and the *PCNA* metagene, as well as KM chi-squared (1 d.f.) statistics for univariate survival analysis based on OS status.

### 5.3. Empirical p-Value Tests of Survival Using Randomly Selected Genes

For each cancer, the single best binarized PC that resulted in the greatest chi-squared statistic during maximum likelihood KM analysis was considered to the be the “observed” test statistic. Recall, this test statistic for the best binarized PC was initially based on individual DNA repair genes whose adjusted gene expression resulted in a significant KM test. Let the number of significant DNA repair genes for a best binarized PC be *p*. We used *B* = 1000 iterations for empirical *p*-value testing. During each *b*-th iteration, a random set of *p* DNA repair genes with the same adjustment to expression were selected, followed by correlation analysis, and then PC extraction via eigendecomposition of the *p* × *p* correlation matrix. Each PC was then binarized and used in KM analysis to determine which PC resulted in the greatest chi-squared test statistic for the set of *p* random genes. After *B* iterations, the empirical p-value was equal to *P* = # {*b*:χ^2(*b*)^ > χ^2^}/*B,* where χ^2^ is the “observed” 1 d.f. χ^2^ test statistic from ML-based KM analysis based on the best binarized PC, and χ^2(*b*)^ is the chi-squared statistic from the best binarized PC extracted from the correlation matrix of *p* randomly selected DNA repair genes used in KM analysis during the *b*th iteration. The bottom of Figure 4 illustrates how the correlation matrix of *p* genes with significant KM analysis were employed to obtain the best binarized PC for predicting OS. 

### 5.4. Genomic Event Rates 

For each cancer, we also summed the number of pathogenic somatic mutations, deletions, and amplifications in the set of 20 driver genes, and in each of the eight groups of DNA repair genes (*DRR, BER, NHEJ, MMR, TLS, DDS, HRR, NER*). Table S3 of the Supplemental Information lists the cancer-specific driver genes used, which we previously reported [45]. Driver gene selection was based on the top 20 driver genes identified by at least two tools in the DRIVERDB database [46]. The GER of each type of event was determined by dividing the sum by the number of genes in the group and the number of tumors obtained for each of the cancers considered. This led to the GER in units of events per gene-tumor. Hierarchical cluster analysis was then used to cluster values of GER for each cancer. Euclidean distance was used as the distance function, while the unweighted pair group method with arithmetic mean (UPGMA) was used for the agglomeration method.

### 5.5. Removing Redundant Genes in Gene Lists

*PCNA* itself was listed as a BER gene and was removed from survival analysis because our primary goal was to remove the genome-wide association of *PCNA* with other genes from the expression of DNA repair genes. For most cancers, *TP53* was listed as a driver gene with high levels of somatic mutations, so it was not included as a DNA repair gene during survival analysis for those cancers. DNA repair genes listed multiple times in the repair pathways described above included *POLD1, POLE, POLH, POLM, WRN, PCNA, LIG1, BLM, BRCA1, FANCA, FANCC*, and *ERCC4*. Only the first occurrence of these genes in their respective pathway lists was used. This resulted in a full list of 123 DNA repair genes, which were used for the univariate statistics presented in Figure 1; and Figure 2. However, after *PCNA* and *TP53* were removed, a final list of 121 unique (non-redundant) DNA repair genes was constructed and used during survival analysis for all cancers. Table S4 of the Supplemental Information lists the DNA repair genes which were excluded from survival analysis because of duplicate listing in the *PCNA* gene list or list of driver genes for the given cancer, and lists driver genes excluded from GER analysis because of duplicate listing in the *PCNA* gene list. 

## 6. Conclusions

In conclusion, our hypothesis-driven focus to target DNA repair gene expression adjusted for the *PCNA* metagene as a means of predicting OS in various cancers resulted in statistically significant sets of DNA repair genes. We also identified that AML, colorectal, and renal papillary cancers may be potentially more opportunistic for inhibition therapy because of less confounding in the form of lower rates of mutations, deletions, and amplifications in DNA repair genes which predict OS in these cancers. The most opportunistic cancer for DNA repair inhibition therapy appears to be AML, since the TCGA cases harbored the lowest rates of somatic mutations, deletions, and amplifications in DNA repair genes.

## Figures and Tables

**Table 1:** DNA repair pathways and 123 genes employed in this study for overall survival (OS) prediction.

DNA Repair Pathway	Genes
Direct reversal repair (DRR)	*ALKBH2, ALKBH3*, *MGMT*
Base excision repair (BER)	*APEX1*, *APEX2*, *APTX*, *FEN1*, *LIG1*, *LIG3*, *MBD4*, *MPG*, *MUTYH*, *NEIL1*, *NEIL2*, *NEIL3*, *NTHL1*, *OGG1*, *PARP1*, *PARP2*, *PCNA*, *PNKP*, *POLB*, *POLD1*, *POLE*, *POLL*, *WRN*, *SMUG1*, *TDG*, *UNG*, *XRCC1*
Non-homologous end-joining (NHEJ)	*DCLRE1C*, *XRCC6*, *XRCC5*, *LIG4*, *NHEJ1*, *POLM*, *PRKDC*, *XRCC4*
Mismatch repair (MMR)	*EXO1*, *MLH1*, *MLH3*, *MSH2*, *MSH3*, *MSH6*, *PMS1*, *PMS2*
Translesion synthesis (TLS)	*POLH*, *POLI*, *POLK*, *POLN*, *POLQ*, *REV1*, *REV3L*
DNA damage signaling (DDS)	*ATM*, *ATR*, *ATRIP*, *BLM*, *BRCA1*, *CCNH*, *CDK7*, *CDKN1A*, *CHEK1*, *CHEK2*, *COPS5*, *DCLRE1A*, *DCLRE1B*, *FANCA*, *FANCC*, *GPS1*, *HUS1*, *MDC1*, *MNAT1*, *MRE11A*, *NBN*, *RAD1*, *RAD17*, *RAD18*, *RAD23A*, *RAD50*, *RAD9A*, *RFC1*, *RFC2*, *RFC3*, *RFC4*, *RFC5*, *TOPBP1*, *TP53*
Homologous recombination repair (HRR)	*BRCA2*, *FAAP24*, *EME1*, *EME2*, *FANCB*, *FANCD2*, *FANCE*, *FANCF*, *FANCG*, *FANCI*, *FANCL*, *MSH4*, *MSH5*, *MUS81*, *RAD51*, *RAD52*
Nucleotide excision repair (NER)	*ERCC8, ERCC6, CUL4A*, *DDB1*, *DDB2*, *ERCC1*, *GTF2H1*, *GTF2H2*, *GTF2H3*, *GTF2H4*, *GTF2H5*, *MMS19*, *RAD23B*, *RPA1*, *XPA*, *ERCC3*, *XPC*, *ERCC2*, *ERCC4*, *ERCC5*

**Table 2:** Kaplan-Meier (KM) logrank test *p*-values for best principal component (best binarized PC) derived from correlation matrix of DNA repair genes with significant individual KM tests after adjustment of expression for age and *PCNA* metagene effects. Cancers listed had only age at diagnosis available in TCGA clinical data.

Cancer	*n*	Kaplan-Meier Logrank *^a^*				Random genes (*B* = 1000) *^b^*			
		A	P	A,P	N	A	P	A,P	N
AML	200	0.0062	0.0018	**0.0157**	0.0062	0.0120	0.0020	**0.0130**	0.0130
Bladder	413	0.0000	0.0000	**0.0000**	0.0001	0.0190	0.0180	**0.0160**	0.0360
Low Grade Gliomas	530	0.0000	0.0000	**0.0000**	0.0000	0.5740	0.2760	0.3880	0.3520
GBM	604	0.0090	0.0005	**0.0231**	0.0008	0.0270	0.0040	0.1600	0.0030
Head & Neck	530	0.0005	0.0009	**0.0006**	0.0008	0.0150	0.1370	0.1120	0.0110
Sarcoma	265	0.0011	0.0032	**0.0008**	0.0001	0.1200	0.0520	**0.0120**	0.0150

A—Expression adjusted for age at diagnosis. P—Expression adjusted for *PCNA* metagene expression. A,P—Expression adjusted for age at diagnosis and *PCNA* metagene expression. N—No adjustment to expression. *^a^*—KM logrank test of binarized principal component scores from correlation matrix of DNA repair genes with significant logrank tests. *^b^*—*p*-Values based on number of times KM logrank test χ^2^ (b) based on randomly selected genes exceeded χ^2^ based on non-random genes. Bold highlighting for *p*-values denotes cancers for which KM logrank or randomization tests were significant when both age at diagnosis and *PCNA* adjustments were made.

**Table 3:** Kaplan-Meier logrank test *p*-values for best principal component (best binarized PC) derived from correlation matrix of DNA repair genes with significant individual KM tests after adjustment of expression for age, stage, and *PCNA* metagene effects. Cancers listed had both age at diagnosis and stage available in TCGA clinical data.

Cancer	*n*	Kaplan-Meier Logrank ^a^						Random Genes (*B* = 1000) *^b^*					
		A	S	P	A, S	A, S, P	N	A	S	A, S	P	A, S, P	N
Breast	1105	0.0057	0.0049	0.0004	0.0069	**0.0015**	0.0060	0.0790	0.0450	0.0050	0.0860	**0.0350**	0.0650
Cervical	309	0.0071	0.0014	0.0015	0.0100	**0.0151**	0.0088	0.2150	0.0630	0.0120	0.2530	0.0730	0.2810
Colorectal	633	0.0550	0.0255	0.0048	0.0188	**0.0106**	0.0330	0.2050	0.0700	0.0120	0.0450	**0.0340**	0.1160
Liver	379	0.0000	0.0002	0.0004	0.0001	**0.0027**	0.0001	0.1150	0.2220	0.0020	0.2140	**0.0130**	0.1720
Lung	522	0.0120	0.0008	0.0003	0.0021	**0.0036**	0.0070	0.4850	0.0560	0.0070	0.1030	**0.0400**	0.3570
Lung SC	505	0.0057	0.0050	0.0040	0.0050	**0.0040**	0.0057	0.0400	0.0290	0.0110	0.0290	**0.0100**	0.0330
Ovarian	603	0.0259	0.0088	0.0184	0.0722	**0.0100**	0.0183	0.2310	0.1250	0.2010	0.5240	0.0700	0.2060
Melanoma	479	0.0000	0.0001	0.0002	0.0000	**0.0001**	0.0001	0.0170	0.0710	0.0190	0.0250	**0.0040**	0.0670
Renal CC	538	0.0000	0.0000	0.0000	0.0000	**0.0003**	0.0000	0.2570	0.4230	0.0100	0.1340	0.2780	0.4710
Renal Pap.	292	0.0000	0.0001	0.0011	0.0000	**0.0012**	0.0000	0.0770	0.0100	0.1060	0.0040	**0.0190**	0.0320
Stomach	478	0.6412	0.0103	0.0180	0.0103	**0.0107**	0.6412	0.6570	0.0070	0.0300	0.0060	**0.0160**	0.6780
Uterine	548	0.0013	0.0024	0.0150	0.0010	**0.0072**	0.0009	0.0250	0.0390	0.3620	0.0070	0.1070	0.0190

A—Expression adjusted for age at diagnosis. S—Expression adjusted for stage. P—Expression adjusted for *PCNA* metagene expression. A,S—Expression adjusted for age at diagnosis and stage. A,S,P—Expression adjusted for age at diagnosis, stage, and *PCNA* metagene expression. N—No adjustment to expression. *^a^*—KM logrank test of binarized principal component scores from correlation matrix of DNA repair genes with significant logrank tests. *^b^*—*p*-Values based on number of times KM test χ^2^ (*b*) based on random genes exceeded χ^2^ based on non-random genes. SC: squamous cell; CC: clear cell. Bold highlighting for p-values denotes cancers for which KM logrank or randomization tests were significant when age, stage, and *PCNA* adjustments were made.

**Table 4:** Multiple-pathway results showing DNA Repair genes whose upregulation or downregulation prolongs overall survival for subjects with RNA-Seq data in TCGA. Cancers listed had significant empirical *p*-value test results (*p* < 0.05). *^a^*

Cancer	Predictors	Upregulation Prolongs OS	Downregulation Prolongs OS
AML *	Genes	*POLN*	*RAD23A, APEX2, EME2*
	Pathways (%)	TLS (100.0)	DDS (33), BER (33), HRR (33),
Bladder *	Genes	*BLM, RAD9A, MGMT, LIG1, MUTYH, DDB1, ERCC5, XPC, FANCD2, MSH5, DCLRE1C, REV1*	*FANCC, ALKBH2, APEX2, LIG3, POLB, GTF2H5, PMS1, PRKDC, REV3L*
	Pathways (%)	DDS (16.7), DRR (8.3), BER (16.7), NER (25.0), HRR (16.7), NHEJ (8.3), TLS (8.3)	DDS (11.1), DRR (11.1), BER (33.3), NER (11.1), MMR (11.1), NHEJ (11.1), TLS (11.1)
Sarcoma *	Genes	*MNAT1, APEX1, APTX, FEN1, NEIL3, DDB1, GTF2H3, FANCI, PRKDC*	*DCLRE1B, POLL, CUL4A, ERCC2, MSH2, FANCG*
	Pathways (%)	DDS (11.1), BER (44.4), NER (22.2), HRR (11.1), NHEJ (11.1)	DDS (16.7), BER (16.7), NER (33.3), MMR (16.7), HRR (16.7)
Breast	Genes	*RAD50, PMS1*	*ATRIP, FANCC, RAD1, RFC3, NEIL3, EXO1, FANCB, FANCD2, FANCI, RAD51, XRCC4*
	Pathways (%)	DDS (50.0), MMR (50.0)	DDS (36.4), BER (9.1), MMR (9.1), HRR (36.4), NHEJ (9.1)
Colorectal	Genes	*DCLRE1C*	*RAD23A, RFC2, POLL, MLH3, FANCL*
	Pathways (%)	NHEJ (100.0)	DDS (40.0), BER (20.0), MMR (20.0), HRR (20.0)
Renal Papillary	Genes	*RAD17, OGG1, DDB2, ERCC2*	*BLM, RAD1, FEN1, LIG1, EXO1, MSH6, BRCA2, EME1, FANCB, LIG4*
	Pathways (%)	DDS (25.0), BER (25.0), NER (50.0)	DDS (20.0), BER (20.0), MMR (20.0), HRR (30.0), NHEJ (10.0)
Lung	Genes	*RAD17, ALKBH3, MGMT, MPG, NEIL1, XPC, LIG4, POLK, REV3L*	*BRCA1, NBN, RAD1, NEIL3, MMS19, FANCI, XRCC5*
	Pathways (%)	DDS (11.1), DRR (22.2), BER (22.2), NER (11.1), NHEJ (11.1), TLS (22.2)	DDS (42.9), BER (14.3), NER (14.3), HRR (14.3), NHEJ (14.3)
Lung Sq. Cell	Genes	*CHEK2, MNAT1, APTX, TDG, FANCE, FANCL*	*XRCC1*
	Pathways (%)	DDS (33.3), BER (33.3), HRR (33.3)	BER (100.0)
Melanoma	Genes	*ATM, MNAT1, MBD4, NEIL1, ERCC5, RAD23B, DCLRE1C*	*MDC1, NBN, MUTYH, POLE, UNG, FANCE, FANCI, POLI, POLK*
	Pathways (%)	DDS (28.6), BER (28.6), NER (28.6), NHEJ (14.3)	DDS (22.2), BER (33.3), HRR (22.2), TLS (22.2)
Stomach	Genes	*CUL4A, POLQ*	
	Pathways (%)	NER (50.0), TLS (50.0)	

*^a^*—Liver cancer not included because it was not a parsimonious model (43 repair genes). * Stage not available, expression adjusted only by age and *PCNA* metagene.

**Table 5:** Qualitative patterns of genomic event rates per gene-tumor for each cluster identified during hierarchical cluster analysis (from Figure 3). Opportunistic cancers for further study in cluster 2 are AML, colorectal, and renal papillary.

Cluster	Cancer	Driver Genes			DNA Repair Genes		
		Mut.	Del.	Amp.	Mut.	Del.	Amp.
1	Uterine *, stomach, bladder, head & neck *, lung, breast, lung sq. cell, liver, cervical *, sarcoma	↑	↑	↑	↑	↓	↑
2	AML, colorectal, GBM *, low grade gliomas *, renal papillary, renal clear cell *	↑	↓	↓	↓	↓	↓
3	Melanoma	↑	↓	↑	↑	↓	↑
4	Ovarian *	↑	↓	↑	↓	↑	↑

* Not significant during empirical *p*-value testing, i.e., DNA repair gene expression adjusted for age, stage, and *PCNA* metagene does not predict OS significantly. ↑: increase; ↓: decrease. Mut: mutation; del: deletions; amp: amplification.

## Supplementary Materials

The following are available online at https://www.mdpi.com/2072-6694/11/4/501/s1, Table S1 of the Supplemental Information lists the 131 *PCNA*-related genes which were available in TCGA expression data. Table S2 lists the multiple linear regression results for each DNA repair gene (dependent variable) and Z-scores for the independent variables: age, stage, and the *PCNA* metagene, as well as KM chi-squared (1 d.f.) statistics for univariate survival analysis based on OS status. Table S3 of the Supplemental Information lists the cancer-specific driver genes used, which we previously reported [45]. Driver gene selection was based on the top 20 driver genes identified by at least 2 tools in the DRIVERDB database [46]. Table S4 of the Supplemental Information lists the DNA repair genes which were excluded from survival analysis because of duplicate listing in the *PCNA* gene list or list of driver genes for the given cancer, and also lists driver genes excluded from GER analysis because of duplicate listing in the *PCNA* gene list. Figures S1–S36 show KM and kernel density plots for acute myelogenous leukemia, bladder, breast, cervical, colorectal, glioblastoma multiforme, head and neck, low grade gliomas, liver, lung sq. cell, lung, melanoma, ovarian, renal clear cell, renal papillary, sarcoma, stomach, and cancers of the uterine.

## References

[1] Hanahan D., Weinberg R.A. (2000). The hallmarks of cancer. Cell.

[2] Curtin N.J. (2012). DNA repair dysregulation from cancer driver to therapeutic target. Nat. Rev. Cancer.

[3] Cannan W.J., Pederson D.S. (2016). Mechanisms and Consequences of Double-Strand DNA Break Formation in Chromatin. J. Cell Physiol..

[4] Forment J.V., Kaidi A., Jackson S.P. (2012). Chromothripsis and cancer: Causes and consequences of chromosome shattering. Nat. Rev. Cancer.

[5] Bryant H.E., Schultz N., Thomas H.D., Parker K.M., Flower D., Lopez E., Kyle S., Meuth M., Curtin N.J., Helleday T. (2005). Specific killing of BRCA2-deficient tumours with inhibitors of poly(ADP-ribose) polymerase. Nature.

[6] Farmer H., McCabe N., Lord C.J., Tutt A.N., Johnson D.A., Richardson T.B., Santarosa M., Dillon K.J., Hickson I., Knights C. (2005). Targeting the DNA repair defect in BRCA mutant cells as a therapeutic strategy. Nature.

[7] Shaheen M., Allen C., Nickoloff J.A., Hromas R. (2011). Synthetic lethality: Exploiting the addiction of cancer to DNA repair. Blood.

[8] Nickoloff J.A., Jones D., Lee S.H., Williamson E.A., Hromas R. (2017). Drugging the Cancers Addicted to DNA Repair. J. Natl. Cancer Inst..

[9] Budzowska M., Kanaar R. (2009). Mechanisms of dealing with DNA damage-induced replication problems. Cell Biochem. Biophys..

[10] Allen C., Ashley A.K., Hromas R., Nickoloff J.A. (2011). More forks on the road to replication stress recovery. J. Mol. Cell. Biol..

[11] Ashworth A., Lord C.J. (2018). Synthetic lethal therapies for cancer: what’s next after PARP inhibitors?. Nat. Rev. Clin. Oncol..

[12] Dedes K.J., Wilkerson P.M., Wetterskog D., Weigelt B., Ashworth A., Reis-Filho J.S. (2011). Synthetic lethality of PARP inhibition in cancers lacking BRCA1 and BRCA2 mutations. Cell Cycle.

[13] Rehman F.L., Lord C.J., Ashworth A. (2010). Synthetic lethal approaches to breast cancer therapy. Nat. Rev. Clin. Oncol..

[14] Gavande N.S., VanderVere-Carozza P.S., Hinshaw H.D., Jalal S.I., Sears C.R., Pawelczak K.S., Turchi J.J. (2016). DNA repair targeted therapy: The past or future of cancer treatment?. Pharmacol. Ther..

[15] Essers J., Theil A.F., Baldeyron C., van Cappellen W.A., Houtsmuller A.B., Kanaar R., Vermeulen W. (2005). Nuclear dynamics of *PCNA* in DNA replication and repair. Mol. Cell. Biol..

[16] Venet D., Dumont J.E., Detours V. (2011). Most random gene expression signatures are significantly associated with breast cancer outcome. PLoS Comput. Biol..

[17] Moldovan G.L., Pfander B., Jentsch S. (2007). PCNA, the maestr of the replication fork. Cell.

[18] Ge X., Yamamoto S., Tsutsumi S., Midorikawa Y., Ihara S., Wang S.M., Aburatani H. (2005). Interpreting expression profiles of cancers by genome-wide survey of breadth of expression in normal tissues. Genomics.

[19] Shimoni Y. (2018). Association between expression of random gene sets and survival is evident in multiple cancer types and may be explained by sub-classification. PLoS Comput. Biol..

[20] Davidson D., Amrein L., Panasci L., Aloyz R. (2013). Small Molecules, Inhibitors of DNA-PK, Targeting DNA Repair, and Beyond. Front. Pharmacol..

[21] O’Cearbhaill R.E. (2018). Using PARP Inhibitors in Advanced Ovarian Cancer. Oncology.

[22] Waks Z., Weissbrod O., Carmeli B., Norel R., Utro F., Goldschmidt Y. (2016). Driver gene classification reveals a substantial overrepresentation of tumor suppressors among very large chromatin-regulating proteins. Sci. Rep..

[23] National Cancer Institute, National Human Genome Research Institute (2005). The Cancer Genome Atlas.

[24] Cerami E., Gao J., Dogrusoz U., Gross B.E., Sumer S.O., Aksoy B.A., Jacobsen A., Byrne C.J., Heuer M.L., Larsson E. (2012). The cBio Cancer Genomics Portal: An Open Platform for Exploring Multidimensional Cancer Genomics Data. Cancer Discov..

[25] Gao J., Aksoy B.A., Dogrusoz U., Dresdner G., Gross B., Sumer S.O., Sun Y., Larsson E., Sander C., Sinha R. (2013). Integrative analysis of complex cancer genomics and clinical profiles using the cBioPortal. Sci. Signal..

[26] Mermel C.H., Schumacher S.E., Hill B., Meyerson M.L., Beroukhim R., Getz G. (2011). GISTIC 2.0 facilitates sensitive and confident localization of the targets of focal somatic copy-number alteration in human cancers. Genome Biol..

[27] Wood R.D. Human DNA Repair Genes. https://www.mdanderson.org/documents/Labs/Wood-Laboratory/human-dna-repair-genes.html.

[28] Wood R.D., Mitchell M., Sgouros J.G., Lindahl T. (2001). Human DNA Repair Genes. Science..

[29] Friedberg E.C., Walker G.C., Siede W., Wood R.D., Schulz R.A., Ellenberger T. (2006). DNA Repair and Mutagenesis.

[30] Lange S.S., Takata K., Wood R.D. (2011). DNA Polymerases and Cancer. Nat. Rev. Cancer..

[31] Ronen A., Glickman B.W. (2001). Human DNA repair genes. Environ. Mol. Mutagen..

[32] Eisen J.A., Hanawalt P.C. (1999). A phylogenomic study of DNA repair genes, proteins, and processes. Mutat. Res. DNA Repair..

[33] Aravind L., Walker D.R., Koonin E.V. (1999). Conserved domains in DNA repair proteins and evolution of repair systems. Nucleic Acids Res..

[34] Strand M., Prolla T.A., Liskay R.M., Petes T.D. (1993). Destabilization of tracts of simple repetitive DNA in yeast by mutations affecting DNA mismatch repair. Nature.

[35] Weber A.M., Ryan A.J. (2015). ATM and ATR as therapeutic targets in cancer. Pharmacol. Ther..

[36] Choi S., Gamper A.M., White J.S., Bakkenist C.J. (2010). Inhibition of ATM kinase activity does not phenocopy ATM protein disruption: Implications for the clinical utility of ATM kinase inhibitors. Cell Cycle.

[37] Erasimus H., Gobin M., Niclou S., Van Dyck E. (2016). DNA repair mechanisms and their clinical impact in glioblastoma. Mutat. Res..

[38] Lawrence M.S., Stojanov P., Polak P., Kryukov G.V., Cibulskis K., Sivachenko A., Carter S.L., Stewart C., Mermel C.H., Roberts S.A. (2013). Mutational heterogeneity in cancer and the search for new cancer-associated genes. Nature.

[39] Tate J.G., Bamford S., Jubb H.C., Sondka Z., Beare D.M., Bindal N., Boutselakis H., Cole C.G., Creatore C., Dawson E. (2019). COSMIC: The Catalogue of Somatic Mutations in Cancer. Nucleic Acids Res..

[40] Li J.F., Konstantinopoulos P.A., Matulonis U.A. (2014). PARP inhibitors in ovarian cancer: Current status and future promise. Gynecol. Oncol..

[41] Dizdar O., Arslan C., Altundag K. (2015). Advances in PARP inhibitors for the treatment of breast cancer. Expert. Opin. Pharmacother..

[42] Sonnenblick A., de Azambuja E., Azim H.A., Piccart M. (2015). An update on PARP inhibitors–moving to the adjuvant setting. Nat. Rev. Clin. Oncol..

[43] Liu J., Lichtenberg T., Hoadley K.A., Poisson L.M., Lazar A.J., Cherniack A.D., Kovatich A.J., Benz C.C., Levine D.A., Lee A.V. (2018). An Integrated TCGA Pan-Cancer Clinical Data Resource to Drive High-Quality Survival Outcome Analytics. Cell.

[44] Bonneville R., Krook M.A., Kautto E.A., Miya J., Wing M.R., Chen H.Z., Reeser J.W., Yu L., Roychowdhury S. (2017). Landscape of Microsatellite Instability Across 39 Cancer Types. JCO Precis. Oncol..

[45] Peterson L.E., Kovyrshina T. (2017). Progression inference for somatic mutations in cancer. Heliyon.

[46] Cheng W.C., Chung I.F., Chen C.Y., Sun H.J., Fen J.J., Tang W.C. (2014). DriverDB: An exome sequencing database for cancer driver gene identification. Nucleic Acids Res..

